# Designing, Implementing, and Evaluating Mobile Health Technologies for Managing Chronic Conditions in Older Adults: A Scoping Review

**DOI:** 10.2196/mhealth.5127

**Published:** 2016-06-09

**Authors:** Nancy Matthew-Maich, Lauren Harris, Jenny Ploeg, Maureen Markle-Reid, Ruta Valaitis, Sarah Ibrahim, Amiram Gafni, Sandra Isaacs

**Affiliations:** ^1^ Aging, Community & Health Research Unit, McMaster University Mohawk College/McMaster University School of Nursing Hamilton, ON Canada; ^2^ Aging, Community & Health Research Unit (ACHRU) School of Nursing McMaster University Hamilton, ON Canada; ^3^ SickKids Hospital mHealth Innovation Toronto, ON Canada; ^4^ Arthur Labatt Family School of Nursing Department of Nursing Western University London, ON Canada; ^5^ Aging, Community & Health Research Unit (ACHRU) Clinical Epidemiology and Biostatistics (CE&B) McMaster University Hamilton, ON Canada

**Keywords:** Telemedicine, Mobile health, Health Plan Implementations, Evaluation Studies as Topic, Design, mHealth Innovations, Frail Elderly, Older Adults, Multiple Chronic Conditions, Home Care Services, Scoping Review, Communication, Information Communication Technologies

## Abstract

**Background:**

The current landscape of a rapidly aging population accompanied by multiple chronic conditions presents numerous challenges to optimally support the complex needs of this group. Mobile health (mHealth) technologies have shown promise in supporting older persons to manage chronic conditions; however, there remains a dearth of evidence-informed guidance to develop such innovations.

**Objectives:**

The purpose of this study was to conduct a scoping review of current practices and recommendations for designing, implementing, and evaluating mHealth technologies to support the management of chronic conditions in community-dwelling older adults.

**Methods:**

A 5-stage scoping review methodology was used to map the relevant literature published between January 2005 and March 2015 as follows: (1) identified the research question, (2) identified relevant studies, (3) selected relevant studies for review, (4) charted data from selected literature, and (5) summarized and reported results. Electronic searches were conducted in 5 databases. In addition, hand searches of reference lists and a key journal were completed. Inclusion criteria were research and nonresearch papers focused on mHealth technologies designed for use by community-living older adults with at least one chronic condition, or health care providers or informal caregivers providing care in the home and community setting. Two reviewers independently identified articles for review and extracted data.

**Results:**

We identified 42 articles that met the inclusion criteria. Of these, described innovations focused on older adults with specific chronic conditions (n=17), chronic conditions in general (n=6), or older adults in general or those receiving homecare services (n=18). Most of the mHealth solutions described were designed for use by both patients and health care providers or health care providers only. Thematic categories identified included the following: (1) practices and considerations when designing mHealth technologies; (2) factors that support/hinder feasibility, acceptability, and usability of mHealth technologies; and (3) approaches or methods for evaluating mHealth technologies.

**Conclusions:**

There is limited yet increasing use of mHealth technologies in home health care for older adults. A user-centered, collaborative, interdisciplinary approach to enhance feasibility, acceptability, and usability of mHealth innovations is imperative. Creating teams with the required pools of expertise and insight regarding needs is critical. The cyclical, iterative process of developing mHealth innovations needs to be viewed as a whole with supportive theoretical frameworks. Many barriers to implementation and sustainability have limited the number of successful, evidence-based mHealth solutions beyond the pilot or feasibility stage. The science of implementation of mHealth technologies in home-based care for older adults and self-management of chronic conditions are important areas for further research. Additionally, changing needs as cohorts and technologies advance are important considerations. Lessons learned from the data and important implications for practice, policy, and research are discussed to inform the future development of innovations.

## Introduction

As developed countries’ populations age and associated chronic health conditions increase, alternatives to hospital and institutional care are needed. The United Nations estimated that by 2050, the world population of older adults over 60 years will have doubled, while the age group over 80 will have tripled from 2013 statistics [1]. In 2013, seniors represented 15.3% of Canada’s population; by 2056, one quarter of Canada’s population will be over 65 years of age [2]. Interest in supporting older adults with chronic conditions to stay in their own homes rather than move to institutions has increased [3]. Consequently, understanding whether mobile health (mHealth) technologies can help to support older adults stay in their homes through improved self-management and increased home care provider efficiency is a priority area of policy development. Given continuous improvements in technologies, it makes sense that mHealth may enhance health care delivery by improving dimensions such as communication, collaboration, and use of evidence-based guidelines for care of older adults with chronic conditions. Although mHealth and technology innovations are rapidly developing, research to guide practice and policy in this arena is still in its infancy. The purpose of this paper is to present a scoping review of the literature pertaining to mHealth solutions intended to address the needs of older adults living at home with chronic conditions. This paper will add further insight into best practices for designing, implementing, and evaluating mHealth solutions for older adults living in their homes and those who care for them (professional and informal caregivers).

It is estimated that approximately one in four older adults have two or more chronic conditions and half of older adults (≥ 65 years) have three or more (ie, heart disease, diabetes, arthritis, chronic lower respiratory tract disease, stroke, chronic obstructive pulmonary disease [COPD], dementia, and hypertension) [4-9]. To manage chronic conditions more effectively, researchers and policy makers have promoted patient and family-centered home-based health care, founded on interprofessional and community-based partnerships [10-15]. Consequently, there is a push to incorporate novel ideas for empowering older adults and their caregivers to manage their own conditions and to foster communication among the circle of care [16-19].

The field of mHealth, as defined by the World Health Organization (WHO) [20] is the “medical and public health practice supported by mobile devices, such as mobile phones, patient monitoring devices, personal digital assistants, and other wireless devices” (p. 6). The growing appeal of mobile solutions for health promotion and health care delivery can be attributed in part to the accessibility of the technology, the level of personalization that technology enables, valuable location-based services, and timely access to information through data, voice, and/or video media [21]. Several studies have piloted mHealth interventions for managing chronic conditions such as diabetes [22-25], COPD [26-28], Alzheimer’s/dementia [29-31], and osteoarthritis [32,33]. Findings from these studies indicate that the use of mHealth interventions has the potential to support successful management of chronic conditions and health behavior change in the areas and systems studied through the following: (1) improving patient self-monitoring and management [34,35], (2) building social networks for patients [36,37], (3) informing health care professionals of patients’ health status [37,36], (4) providing indirect feedback interactions [38,39], (5) tailoring care and education to patient needs [40-42], and (6) improving communication among health care professionals [43,44]. It is anticipated that this list will perpetually expand considering the recent momentum of mHealth innovation.

Despite these promising opportunities, the current literature supporting the use of mHealth primarily includes pilot and/or feasibility studies [45]. Large-scale trials and information on best practices for the design, implementation, and evaluation of such technology are limited. A recent survey by the WHO found that only 12% of member states reported evaluating mHealth services and that little was known about how to effectively evaluate such solutions [20]. Concerns related to data security, technology literacy levels of potential end-users, and other key issues highlight the importance of considering the overall architecture of the mHealth system and the context in which it will be used. As a result, there is a need for evidence to inform the successful research and development of mHealth solutions and to garner an improved understanding of the key elements and fundamental components of designing, implementing, and evaluating successful mHealth applications for managing chronic conditions associated with a community-dwelling aging population [20,46]. A scoping review of the literature is presented as a fundamental first step to understanding the current practices, state of knowledge, and evidence to inform future directions. As per the convention in conducting scoping reviews [47], this paper does not systematically evaluate the methodological rigor of included studies.

## Methods

### Study Design

The purpose of scoping reviews includes comprehensively synthesizing evidence to map a broad, complex, or emerging field of study and to identify gaps with the intent to inform practice, policy, and future research [47]. A scoping review methodology was chosen as a tool for systematically mapping and disseminating the breadth of research available to address the broad, complex, and novel research question, “What are the current practices and recommendations for designing, implementing, and evaluating mHealth solutions to support older adults with chronic conditions living in their homes?” More specifically, this review considers solutions that enable care-related communication, information sharing, and information access for older adults and those involved in the care of older adults with chronic conditions living in their homes. This includes technologies intended for use by health care providers (HCPs), family, significant others, and friends involved in care, as well as older adults themselves. HCPs include all health professionals as well as unregulated providers such as personal support workers. This review, as is appropriate with a scoping review methodology, encompasses a broad range of study designs and varied contexts including different countries, communities, technologies, implementation milieus, and chronic conditions/stages without separating those with single conditions from those with multiple chronic conditions.

To complete this scoping review, we followed the five-stage framework developed by Arskey and O’Malley [47] and further defined by Levac, Colquhoun, and O’Brien [48]. The stages include: (1) identification of the research question, (2) identification of relevant studies, (3) selection of relevant studies for the review, (4) charting information and data from the selected literature, and (5) summarizing and reporting the results of the review.

### Identification of the Research Question

The research question was identified from a preliminary scan of the literature and drawing on the expertise of the research team and several stakeholders. Rationale for the question arose from the lack of existing consensus in the academic literature on designing, implementing, and evaluating mHealth solutions in community-based settings, specifically as it pertains to older adults living at home with chronic conditions.

### Identification of Relevant Articles

The team collaboratively planned and implemented a search strategy to identify relevant literature that was specific to mHealth solutions targeting chronic conditions and community-based care of older adults. Keywords and related subject headings were identified in consultation with research librarians in order to capture a comprehensive list of potential sources. Keywords were identified and combined to address three components of the research question: (1) mobile or electronic devices, (2) technology-based health care delivery, and (3) an aging population and/or chronic conditions (Textbox 1). Keywords were searched using Boolean operators. The databases used to locate the relevant literature were the following: Cochrane Library, Embase, PsychInfo, Medline, and CINAHL. Databases were searched for English language articles published between January 2005 and March 2015. In addition, reference lists were searched and a key journal hand search was completed (Journal of Medical Internet Research). Only articles published in peer-reviewed scientific journals were considered for review.

**Scoping review keyword search strategy**

Mobile/Electronic Device

- Cellular Phone, Mobile Phone, PDA, Smartphone, Tablet

Mobile Health/Telehealth

- Computer interface, Design, eHealth, Human factors, Implementation, Integration, mHealth, Mobile Health, Telecare, Telecommunication, Telehealth, Telemedicine, Usability, User-centred design, User-friendly

Condition/Population

- Cerebrovascular Accident, Chronic Disease, Community, Disease Management, Health Program, Health Service, Healthcare Delivery, Home care, Inter-professional, Point-of-care, Quality of Life, Rehabilitation, Rehabilitation Care, Reintegration, Stroke

### Selection of Relevant Articles for the Review

Two reviewers independently searched the titles and abstracts of the retrieved literature. Conflicts were resolved by a third reviewer and through team consensus. Inclusion criteria were mHealth technologies focusing on at least one of the following: (1) chronic conditions associated with aging populations, (2) HCPs providing home care, and/or (3) older adults living at home and/or their informal caregivers. Research articles using different methodologies (qualitative, quantitative, and systematic reviews) as well as theoretical papers were included and all papers had to be in English. The exclusion criteria were the following: (1) mHealth solutions being used for diagnostics/imaging, acute care, body and environment monitoring or support devices, or robotics; (2) technology pertaining to healthcare in developing countries; and (3) non-English language publications. Methodological quality of the published articles was not a criterion for exclusion/inclusion. This enabled the inclusion of a breadth of knowledge pertaining to the research question, as is consistent with scoping review practices [47-49].

Articles that potentially met inclusion criteria through abstract review were reviewed in full by team members. Meetings were held regularly to discuss reviewers' decisions specific to the inclusion and/or exclusion of articles. Both inclusion and exclusion criteria were revised as the search evolved, in order to best address the research question. Under the final revised criteria, only articles pertaining to older adults (>50 years old) with one or more chronic conditions living in their homes were included.

### Charting, Summarizing, and Reporting the Results of the Review

A descriptive-analytical narrative method was used to extract and chart the data from the selected articles [47-49]. Using the same process of team consultation, data from the selected articles were first extracted onto a data charting form developed by the research team using an iterative process. Charts were used to collate, summarize, and share data for team review and decision making. Data entered included the following: authors, year of publication, purpose of the paper/study and innovation, study location and context (setting, end-users of innovation), study design, outcomes measured, main findings, and lessons learned. A coding scheme (framework) was created under four thematic categories: (1) design and development, (2) implementation, (3) evaluation, and (4) risks and benefits. Full articles were imported as pdf files into NVivo 10, a software program for qualitative analysis, for more detailed data extraction and coding. The authors applied the coding scheme to all pertinent text, and then further coded the data under emergent themes using an iterative process.

## Results

### Selection and Characteristics of Source Documents

**Figure 1:**
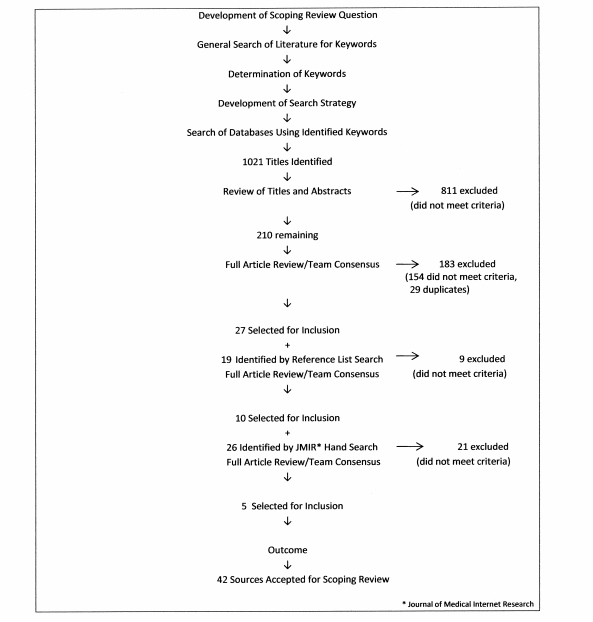
Search strategy and results.

In total, 1021 published articles were identified in the database search (Figure 1) and of these, 811 were excluded based on a review of titles and abstracts. Of the remaining 210, another 183 including 29 duplicates were excluded through independent review followed by team reviewer consensus, leaving a total of 27 articles. Ten more articles found through reference list reviews plus 5 articles found through a manual search of a key journal (Journal of Medical Internet Research) were accepted after applying inclusion/exclusion criteria. A total of 42 sources were included in this review.

Of the 42 studies included (Table 1), 9 were from the United States, 5 from Canada, 5 from the United Kingdom, 6 from Scandinavian countries (Denmark=3, Norway=1, Finland=1, Sweden=1), 3 from the Netherlands, 4 from Australia, 2 from New Zealand, 4 from East Asia (South Korea=1, Japan=1, Taiwan=2), and 1 each from China, Italy, Belgium, and Poland.

Seven of the selected articles were theoretical papers (discussion and position papers). Four were descriptive reports of existing interventions, and 3 were case studies describing processes of mHealth implementation. Two articles described predictive modeling techniques for screening patients in use of technology. Three qualitative descriptive studies elicited opinions concerning mHealth. There were 13 papers in which mHealth solutions were evaluated; 6 controlled trials, 3 mixed-methods studies, and 4 qualitative studies. Three studies were cross-sectional surveys, 3 were systematic or scoping reviews, 2 were methods papers, and 1 paper focused on simulation.

Of the 42 articles, 17 focused on older adults with single chronic conditions: diabetes (n=4), stroke (n=5), heart condition (n=4), COPD (n=1), and dementia or cognitive impairment (n=3). Six articles involved older adults with any chronic condition or multiple chronic conditions. Conditions were not specified in 18 articles, in some cases referencing older adults (n=5) or home care patients (n=6) and caregiver burden (n=1).

The majority of mHealth solutions described were designed for use by both patients and HCPs (n=19), followed by HCPs only (n=7), patients only (n=5), caregivers, patients, and HCPs (n=4), and patients and caregivers (n=3); one mHealth solution was targeted exclusively at family caregivers. The remaining 3 articles were nonspecific.

**Table 1:** Review article characteristics.

Article Location	Article Type^a^	Type of Article/ Study Design	Condition	Innovation	Innovation End-users
Alpay et al (2010) Netherlands [88]	1	Discussion paper	NS^b^	eHealth patient empowerment	Patients
Bujnowska-Fedak & Mastalerz-Migas (2015) Poland [82]	4	Cross-sectional survey	NS	Internet use for health by older adults	Patients
Barakat et al (2013) USA [67]	2	Qualitative descriptive	NS	eHealth competencies /HCP^c^ workshop participants	HCP
Blake (2008) UK [90]	1	Discussion paper	Chronic disease	Mobile technology for monitoring & health promotion	Patients and HCP
Bosl et al (2013) USA [89]	2	Predictive modelling	NS	HCP screening for medication compliance at home	HCP
Boulos et al (2011) UK [53]	1	Discussion paper	NS	Mobile phones and app technology for mHealth	Caregivers, patients and HCP
Chan et al (2012) Australia [76]	2	Descriptive report	Diabetes	Web-based SMS^d^ /mobile terminal	Patients and HCP
Chiang et al (2012) Taiwan [59]	3	Nonrandomized quasi- experimental design	Caregiver burden	Telemonitoring/phone counseling	Caregivers
Chumbler et al (2012) USA [84]	3	Single-blind RCT^e^	Stroke	Text messaging, phone, home visits	Patients and HCP
Cicolini et al (2014) Italy [56]	3	RCT	CVD^f^	Text messaging reminders	Patients and HCP
Dale et al (2014) New Zealand [77]	3	Mixed-methods survey; Pre-post test pilot	CVD	Mobile phone & Internet system	Patients and HCP
Eland-de-Kok et al (2011) Netherlands [91]	5	Systematic review	NS	eHeath vs usual home care	Patients and HCP
Esser & Goossens (2009) Netherlands [62]	1	Literature review/ theoretical	NS	User-centered design framework	Patients and HCP
Forducey et al (2012) USA [54]	3	Controlled trials (2 randomized, 1 not) (pilot studies)	Cognitive impairment	Telehealth: text messaging, videophone, phone	Caregivers, patients and HCP
Hall et al (2012) USA [78]	1	Discussion paper	NS	Telemedicine and mHealth for older adults	Patients
Hebert et al (2006) Canada [65]	1	Implementation decision framework	Diabetes and chronic diseases	Telecare implementation	Patients and HCP
Huang & Hsu (2014) Taiwan [58]	3	Qualitative pilot	NS	Social networking & telehealth; tablets	Caregivers, patients and HCP
Huijbregts et al (2009) Canada [85]	3	Mixed methods	Stroke	Telehealth delivery system	Patients and HCP
Joubert et al (2013) Australia [55]	5	Literature review	Stroke	Telestroke	Patients and HCP
Kim et al (2012) South Korea [42]	3	Quasi-experimental design intervention study	COPD^g^	uHealth devices (monitoring, education/home visiting)	Patients and HCP
Malinowsky et al (2014) Sweden [57]	3	Case control	Cognitive impairment	Tech screening tool	Patients
May et al (2011) UK [87]	2	Qualitative descriptive	Chronic disease	Telecare implementation	Caregivers, patients, and HCP /managers
McCullugh et al (2013) UK [51]	2	Case review	NS	Telehealth evaluation framework	Patients and HCP
Nielsen & Matthiassen (2013) Denmark [73]	2	Case study	NS	mHealth implementation and home care	NS
Nielsen & Mengiste (2014) Denmark [72]	2	Case study	NS	Mobile health diffusion (social world theory) and home care	NS
Nundy et al (2012) USA [52]	3	Qualitative descriptive pilot	Diabetes	Text messaging with follow-up	Patients and HCP
Nyborg et al (2013) Denmark [64]	2	Descriptive report	NS	Mobile phone for nurses and home care	HCP
Pandey et al (2013) USA [61]	4	Cross-sectional survey	Stroke	Mobile phones and app technology	Caregivers and patients
Paré et al (2011) Canada [74]	3	Mixed methods	NS	Laptop computer software and home care	HCP
Saywell et al (2012) New Zealand [79]	6	Study protocol/mixed methods	Stroke	Telerehab program	Patients and HCP
Stroulia et al (2012) Canada [50]	3	Qualitative/ ethnography	NS	Mobile ICT and home care	HCP
Townsend et al (2013) Canada [92]	2	Qualitative descriptive	Chronic conditions (multiple)	Ethics of eHealth	Caregivers and patients
Van Hoecke et al (2010) Belgium [75]	2	Descriptive report	Diabetes and multiple sclerosis	Web-desktop with PDA interface	Patients and HCP
Varnfield et al (2011) Australia [81]	2	Descriptive report	CVD cardiac rehab	Mobile phone and internet video conferencing	Patients and HCP
Varsi et al (2013) Norway [70]	3	Qualitative descriptive	NS	Internet patient provider communication service	Patients and HCP
Vuononvirta et al (2011) Finland [80]	2	Qualitative descriptive	NS	TeleHealth compatibility	HCP
Walters et al (2010) Australia [35]	6	Study protocol/RCT	CVD cardiac rehab	Mobile phone platform	Patients and HCP
Wang et al (2014) China [71]	5	Integrative review	Chronic disease	Mobile phone apps	Patients
Yellowlees (2005) USA [68]	1	Position paper/ theoretical	NS	Principles of successful telemedicine	NS
Zhang et al (2008) Japan [86]	6	Simulation testing	NS	Mobile phone & Internet; Teleconferencing and home care	HCP
Zhang et al (2014) UK [60]	2	Predictive modelling	Dementia	HCP screening for use of video streaming by patients	Patients and HCP
Zulman et al (2013) USA [83]	4	Cohort study - sample survey	Chronic conditions	mHealth technology for out-of-home caregiving	Caregivers and patients

^a^Type of Article: 1=theoretical, 2=descriptive, 3=intervention study, 4=population/cohort study, 5=review, 6=other

^b^NS: nonspecific

^c^HCP: health care providers

^d^ SMS: short message service

^e^RCT: randomized controlled trial

^f^CVD: cardiovascular disease

^g^COPD: chronic obstructive pulmonary disease

### Review Findings

Results pertaining to mHealth solutions are organized under 3 phases of development: design, implementation, and evaluation. Given the iterative and cyclical nature of designing, implementing, and evaluating mHealth technologies, these categories are not discrete entities and inevitably overlap. The categorical terms were used to organize the review findings as commonly presented in the papers reviewed. Each section is discussed within thematic constructs derived from the analysis of the selected literature using an iterative process of qualitative content review.

### Designing mHealth Solutions

Two thematic constructs emerged from the literature pertaining to practices and considerations in designing mHealth solutions: (1) user-centered design and (2) interdisciplinary/collaborative team approaches.

#### User-Centered Design

Recommendations from both research findings and theoretical perspectives are consistent regarding the need for end-user design. Multiple examples of end-user design approaches were provided within the literature [46,50-52]. End-user engagement throughout the design and development process guided researchers and developers in designing solutions to be acceptable, feasible, and sustainable by fitting within the end-user’s context [48]. The user-centered approach allowed researchers to obtain feedback from patients, caregivers, and HCPs who will be using the solution to address their specific needs and ideas, taking into account technology literacy and personal preferences [50,53-56]. Consideration of technological literacy and acceptance was particularly important when the mHealth solution involved older adults with cognitive impairment [56-58]. Attention to HCP aptitudes and preferences for technology as well as HCP value-based practices and adherence to patient-centered care were also deemed necessary [51,54,58]. Further, mHealth solutions are needed that address the health and information needs of informal caregivers related to their family member’s well-being, with reassurances that health concerns are being managed [54,59-61]. Examples of design features solicited to support end-user needs and preferences are presented in Textbox 2.

**User-centered design features.**

Software/App Features

- Graphs displaying patient-related trends (ie, glucose monitoring and medication) [58,75,89]
- Notification system, which alerts agencies, case managers, and professionals of specific patient responses that require attention and follow-up [52,75]
- Text messages (short message service, SMS), which contain motivational and educational information as well as reminders to improve treatment adherence in chronic diseases [35,71,81]
- Video messaging (patients with dementia) [60]
- Client management features: scheduling [75], patient record/information access [64,72,83], voice and text messaging [64,73]
- Aids for seniors: vision, hearing, memory [53]
- Patient texting features for reporting health status [76,85]

Hardware/Mobile Devices

- Mobile devices with large touch-screens and large virtual buttons (vs hard buttons) [53]
- Mobile phones not requiring end-users to reboot the system frequently; minimizing pop-ups; remote, seamless maintenance [53]
- Lighter tablets with a touch pen to suit the mobility of homecare providers [86]
- Voice input function [58]
- Cloud computing resources [58]
- Smartphones and voice-over-Internet protocol software applications (eg, Skype) [78]

Esser and Goossens [62] discuss the need for a user-centered design framework when designing mHealth solutions to meet the needs of an aging society, specifically through telemedicine. Their framework is derived from a review and consolidation of established frameworks used within the information and technology industry. The framework promotes patient-provider interaction as the starting point for design, acknowledging that “patient-provider consultation is considered to be the most complex, due to the interpersonal relationships that are involved” (p. 33). Other constructs incorporated into their model include technology acceptance and technology-mediated communication.

#### Interdisciplinary/Collaborative Team Approaches

Reviewed literature consistently reported the use of interdisciplinary team-based approaches in the process of designing and developing mHealth solutions. The interdisciplinary team in this literature consisted of technology experts and health care professionals as well as end-users and other affected stakeholders [46,51,52]. The literature identified the need for technical experts to work collaboratively in an iterative design process with patients, caregivers, health care professionals, and key stakeholders vested in health care delivery [51,53,63,64]. Collaborative practices enabled the documentation of a user-accepted yet technically feasible list of user requirements. One such example was the inclusion of features of minimal complexity for end-users, which were still based on the most advantageous and available technologies for designing solutions [51]. At the initial stage of design, Esser’s user-centered design framework recognized the importance of three forms of input: individual, organizational, and technical context [62]. This latter statement supports the notion of multi-stakeholder/ multi-sectoral involvement as a means of ensuring different stakeholder interests are met [46].

In summary, continued engagement with end-users as well as collaborative team approaches that encourage multiple stakeholder involvement, are both essential in the successful design and development process for mHealth solutions. User-centered approaches enable researchers and engineers to prioritize an understanding of the context in which the solution will be used by a diverse group of end-users. It also helps to establish early on the specific app features and hardware considerations perceived to be acceptable, preferable, and compatible with the needs of the end-users. Integrating these features and hardware considerations throughout the design and development phase of the solution is imperative, as it influences end-users' response, engagement, uptake, and adherence. Collaboration among stakeholders ensures different interests are appreciated, and that knowledge transfer between content and technology experts is maximized.

### Implementing mHealth Solutions

Three thematic constructs emerged from the literature pertaining to successfully implementing mHealth solutions: (1) feasibility in relation to organizational and systems readiness, (2) acceptability of the mHealth solution, and (3) usability in relation to the different end-users. These factors were reported to either facilitate or hinder the implementation of mHealth solutions.

#### Feasibility: Organizational and Systems Readiness

The need for health system readiness to adopt mHealth solutions was highlighted in much of the theoretical literature. At the institutional level, financial resources, policies, and workplace culture all play a key role in the successful adoption of mHealth technologies [46,65]. Organizational readiness for adoption was recognized as a multi-faceted and dynamic construct, essential for driving change [66]. Compared to most other industries, health care is relatively slow to adopt new technology and such resistance to change has likely contributed to the limited widespread adoption of mHealth solutions beyond the pilot phase [51,53,65,67]. The literature refers to the inertia and resistance to change that can exist within organizations [46,65], further highlighting the importance of a strategic business-focused plan for implementation [68]. The strategic plan needs to ensure sufficient, sustainable funding for the costs associated with implementing and maintaining the solution [46,52,67]. A look to business models for designing long-term management and support [68] could also inform research studies and development initiatives, currently limited in scalability beyond the pilot phase [46,69].

May et al reported how general uncertainties about policies and management systems were to blame for the lack of successful uptake of telecare services [46]. They argue for a systems perspective based on normalization process theory to ensure successful mHealth (telecare) implementation. In this approach, all stakeholders are involved during development and implementation to include the different end-user groups, as are suppliers/developers, policy makers, and health care managers. Further to this, Herbert et al proposed a decision framework when implementing telehealth solutions in chronic illness care, taking into account factors such as associated disease burden, health care patterns and resources, evidence of success, and overall readiness (management, service, and delivery) [65].

A lack of a clear reimbursement schedule was described as a barrier for clinicians to adopt mHealth technology [54,62,70]. A cohesive implementation team with clear leadership, ownership, and accountability was recommended to mitigate these uncertainties and facilitate acceptance by stakeholders. Further, choosing clinician champions who feel they have ownership of the system could effectively facilitate user acceptance within an organization [68]. Institutions were found to facilitate successful implementation by providing effective, ongoing technical and professional support to HCPs as end-users [71]. In the words of Yellowlees, “train, train, and train again” [68].

The process of adoption and diffusion of an mHealth solution was reported in a case review from Denmark [72]. Adoption of telehealth for community-dwelling older adults at a national level was reportedly triggered by demonstrated successes of a municipal project that simultaneously met the interests of major government stakeholders looking for fiscal efficiencies in health care delivery. The process was described as rapid diffusion accepted by government and then driven downward. With resistance felt at the micro level while local systems and the workforce adapted, the eventual target of full diffusion was reached after 10 years. The authors propose a social world perspective that offers an analysis of the politics of sociotechnical change applied at the macro (governance and finance), meso (manager), and micro (end-user) levels of experience. Their model speaks to differences in cultures and interests of different professional sectors, emphasizing the need for health care and technology to find a “common vocabulary” in order to enable successful mHealth implementation.

#### Acceptability of the mHealth Solution: The End-User Perspective

Delays in local adoption of mHealth technology were attributed to top-down approaches that neglect to address the impact on workload adjustments and practice preferences by the end-user workforce [72]. Not surprisingly, health care providers working in the community were more likely to adopt new technology if they saw benefits in terms of professional role support [67,72]. It was not uncommon for health care providers to report negative perceptions of the solution, specifically viewing it as a tool for organizational micromanagement [72,73]. This perception significantly reduced their willingness to adopt new technology. Generally, the mobile solution was accepted more by HCPs and patients when it had the capability to be customized to both the population of interest [74,75], and to individual preferences and response needs [52]. For example, when end-users associated automated alert messaging with responsive follow-up, they reported higher interest in engaging with the solution [52]. Studies have reported on the unique qualities of using mobile interfaces for health care purposes [58]; when information was found to be too complex to be read on a mobile screen, the information was not accessed effectively or at all [73].

#### Usability: User-Technology Interface

The perceived value and ease-of-use by the end-user was identified as a critical factor in successful adoption of an mHealth solution. End-user preferences and levels of technical literacy were felt to affect the way health care information is shared and accessed [57,67]. Generally, a solution will not be used if it is perceived to be “more trouble than it is worth” [67,72]. This was evident in situations where the solution was considered to be too time-consuming [76], unreliable [67], or generally burdensome (eg, multiple passwords to remember, difficulty with software installation) [58,77]. Solutions that are easily adoptable must fit naturally into the existing context, whether that means into the health care providers’ or patients’ existing daily workflow and routines [58,68] or integrating with other existing tools and applications [58,76,78]. For example, an application well received by end-users was developed using Facebook as the platform, enabling older adults and their family members to review health status information using familiar social network technology, while HCPs were able to access selected information related to patient care [58].

In addition, special consideration must be given to the varied types of information that are shared via different mobile devices; mobile phones are limited in the amount of information that can fit on a screen compared to computers [53]. HCPs preferred larger computer screens over mobile devices for recording patient information [73]. From a patient perspective, tablets with touchscreens may be more accommodating for individuals with limited vision and dexterity, compared to mobile phones [58].

In summary, factors that support or hinder implementation of mHealth solutions include the following: (1) institutional environment such as culture, policies, and readiness to change; (2) the availability of a comprehensive business plan; (3) personal factors of the different end-users including perceived value of the mHealth solution; and (4) factors related to the solution itself, for example, ease-of-use by different types of end-users. These data highlight the importance of researchers understanding the culture, values, and readiness of different stakeholders and end-users from project inception, and also to continue to monitor and address end-user and stakeholder feedback.

### Evaluating mHealth Solutions

A variety of quantitative, qualitative, and mixed-methods designs were used to evaluate mHealth solutions for older adults living at home. Researchers were interested in evaluating aspects of application design and implementation (eg, feasibility, acceptability, and usability), as well as health outcomes experienced by clients receiving the interventions (Table 2). Selected approaches used to evaluate mHealth solutions were grouped under 3 thematic constructs: (1) design and formative evaluation; (2) implementation, process, and outcome evaluation; and (3) frameworks for planning evaluation.

**Table 2:** Constructs measured in mHealth studies

Domain	Construct	Measurement Tools
**End-User Perceptions: Acceptability & Feasibility**	End-User Satisfaction	*Clients:* Written questionnaire survey post-intervention [35, 42, 77, 81, 86] Interviews [58, 79] Focus groups post-intervention [85] *HCPs:* Structured questionnaire survey post-intervention [74, 81] Focus Group [85] Semi-structured interviews [74] *Family caregivers:* Interviews [58]
	Usability	*Frequency of use and usage patterns:* Measured by built-in data analystics system[58, 81] *Ease of use:* Questionnaires [35] Interviews [58] Technology Usability Scale [57]
	Intervention Feasibility	Attendance/utilization rates [77,85] Focus group [85] Facilitator log [85]
**Patient Health Outcomes**	Quality of Life/Well-being	Reintegration to Normal Living Index [85] Functional Independence Measure (Telephone version; Motor subscale) [84] Late-life Function and Disability Instrument [84] Geriatric Depression Scale [85] Kessler 10 [35] Diet Habits Questionnaire [35] EuroQol’s EQ-5D [35] Morbidity (hospital readmissions) and Mortality obtained by hospital records [35] Heart Healthy Eating [77] Heart Healthy Eating Self-Efficacy Scale (HHESES) [77]
	Condition-Specific Disease Severity	*Stroke/Cardiovascular:* Stroke-Adapted Sickness Impact Profile [85] Stroke Impact Scale [79] Stroke Self-Efficiacy Questionnaire [79] Cardiac Rehabilitation ssessment Tool [35] Chedoke-McMaster Stroke Assessment Activity Inventory [85]
	Physical Function	Grip Strength (Jamar handheld dynamometer) [79] Step test [79] Active Australia Survey [35] Walking activity measured by pedometer [35] 6-Minute Walk Test [35] Berg Balance Scale [85]
**Other Outcomes**	Patient Treatment Adherence	Self-report [35] Dropout rate, obtained from trial recruitment spreadsheet [35]
	Caregiver and Family Well-being	Caregiver Burden Inventory [59] Feetham Family Functioning Survey [59] Mastery of Stress Scale [59]
	Goal Attainment	Goal Attainment Scaling [85]
	Cost Effectiveness	EuroQol - 5D [79] Reported costs of staff time, equipment and facility costs (from hospital’s financial database), cost estimates for other technology costs at current market value [35]

#### Design and Formative Evaluation

Studies that addressed design features with respect to acceptability and usability tended to use qualitative data collection strategies: focus groups, in-depth and semi-structured interviews [42,51,52,70,74,79,80], persona-based scenarios [50,51], as well opinion surveys using structured questionnaires [42,61,81-83]. Functionality (usability/feasibility) was tested within simulation environments [50,60]. A good understanding of the unique needs and characteristics of the end-users was obtained through the use of interviews, observations, and focus groups [51]. End-users included clients, family caregivers, and HCPs (Table 2).

#### Implementation, Process, and Outcome Evaluation

Several methods and data collection strategies were used during pilot and small scale implementation studies to evaluate mHealth solutions used in the context of home and self-management for older adults. Studies investigating the adoption and implementation of mHealth solutions captured end-user utilization statistics through self-report [83] or automated data-generating features of the application itself [77,81]. Case studies including document review and key informant interviews were used to describe implementation across a health care system [72,73]. Opinions concerning barriers and facilitators to implementation were again captured through interviews and opinion surveys with key informants and end-users [46,74,80,81]. Costs of implementation were assessed using cost-benefit analysis techniques [35]. Core competencies in eHealth for HCPs were identified through a facilitated process with workshop participants [67].

Controlled trials assessing health outcomes associated with the use of mHealth solutions frequently incorporated standardized tools or scales designed for the specific health outcome or chronic condition of concern [59,79,84,85]. Parametric measures (eg, blood pressure, BMI, glucose levels) were used to determine changes or differences in health status [35,55,76]. One systematic review investigated the overall effectiveness (ie, cost, satisfaction, and quality of life) of eHealth using the Internet as a mechanism for interactive communication (instruction, information, monitoring) between patients and professional care providers [91]. Another review investigated the benefits of mobile phone interventions for long-term chronic condition management [71].

#### Frameworks for Planning Evaluation

McCullagh et al discussed phases of evaluation: (1) formative evaluation, conducted during application design and prototype testing of mHealth solutions in the self-management of chronic conditions; (2) summative evaluation, conducted during limited launches and pilot phases; and (3) population outcome evaluations, applied to full implementation after pilot phases, to determine the impact of complex interventions embedded in health care delivery systems [51]. The need for a common evaluation framework was identified, which incorporates all phases of mHealth solution development and supports an iterative process of development and knowledge transfer between developers, health care experts, and end-users. Similarly, Dale et al, in their work on mHealth solutions to support self-management by cardiac patients, followed a stepped process of evaluation starting with conceptualization, followed by formative research, and pre-testing to be followed by outcome evaluation through randomized controlled trials [77]. Each evaluation phase addresses different purposes in the development process.

In summary, methods used to evaluate mHealth solutions varied across the literature, including a variety of quantitative and qualitative data collection strategies and tools. Standardized tools were used for targeted outcomes of interest, and were often tailored to the chronic condition or client population under study. Other outcomes were more specific to reported behaviors and body metrics. In most cases, studies were either feasibility or pilot investigations, offering limited knowledge concerning the impact of full-scale implementation [51]. Researchers acknowledge the need for evaluation frameworks to guide a process of evaluation that follows the different phases of mHealth development and implementation from pilot studies to full-scale implementation [51,77].

## Discussion

The purpose of this scoping review was to identify current practices and recommendations in designing, implementing, and evaluating mHealth technologies to support older adults and their caregivers in managing their chronic conditions while living at home. Lessons learned from this review are highlighted in Table 3 as they apply to the mHealth development process and from these, specific recommendations are offered. The lessons learned and recommendations will contribute richly to future mobile health developments for this rapidly growing population and technological context.

**Table 3:** Lessons learned in designing, implementing, and evaluating mHealth to support older adults with chronic conditions at home.

Design, Implementation, and Evaluation Domains	Recommendations
A good understanding of the end-users’ context is critical	Engage end-users in activities such as personas and scenarios or simulations [50,53] with the technology; Involve app users and stakeholders early and often in the design process [46,88]; Consider universal design and accessibility principles to include engagement from end-users with a variety of abilities and needs [54]; Design apps that adapt to HCP’s or patients’ existing daily workflow and routines [58,68]
Less can be more on a mobile interface	Minimize navigation screens to two [53]; Include features with minimized complexity for end-users that are still based on the most advantageous and available technologies [51]; Match complexity and length of messaging to screen size for digestibility and readability [73]
Develop a strategy for interprofessional collaboration (ie, health care and technical expertise)	Create interdisciplinary development teams that consist of technology experts and health care professionals along with end-users and other affected stakeholders [46,51,52]; Ensure ongoing communication/sharing of ideas between health care and IT experts to enable successful implementation [72]
System and service reliability is essential for successful implementation	Be aware that malfunctions can cause frustration and negative perceptions of the solution [53]; Carefully design training approaches tailored to the needs of the end-users [50,71]
Look to business models for designing long-term maintenance and support	Carefully and realistically consider funds and timeline when planning for implementation [54]; Incorporate ongoing support and hardware maintenance/upgrades into budget [50]; Employ sound business models to secure investment from key government stakeholders [72]
Assemble a cohesive implementation team	Acknowledge that buy-in from both internal (end-users) and external (administrators/management) stakeholders is important [54]; Call on clinician champions as drivers to support the use of the solution [68]
An evaluation plan should be considered early on	Use an evaluation framework that incorporates all phases of mHealth application development [51]; Follow a stepped process of evaluation starting with conceptualization; consider a plan that will enable long term impact evaluation and costing [77]

### Implications for Policy and Future Development

The findings from this review have implications for all stakeholders including researchers, clinicians, homecare providers, software developers, patients, and their families. In one of the few widespread technology implementation studies in community health care, it was clear that the extent of technology adoption was related to the end-users’ perceived value or perceived risk of using the technology [73]. This supports one of the core constructs of normalization process theory pertaining to coherence or sense-making of the innovation [87]. It is therefore vital that clinicians, researchers, and mHealth designers consider hardware and software factors in the context of end-users’ needs, preferences, and activities to ensure the solution is working for the user and not the other way around. Developers should strategically put together a team that has the capacity, including knowledge, skills, and resources to implement and maintain mHealth solutions, and who can speak to the specific needs of HCPs, patients, and their family supporters.

In only a few of the articles reviewed, were considerations and findings guided and presented within a theoretical framework [46,51,62,77]. The use of a framework enabled an approach that acknowledges the complexity of mHealth development when involving a diverse set of stakeholders and their interests in the midst of dramatic change in health care delivery. The magnitude of this challenge becomes more acute when we recognize the different levels of support required for older adults living at home—from total independence with the option to use mHealth technology as desired, to a gradation of dependency requiring the involvement of informal caregivers and health care providers. Further, the development of mHealth solutions presents its own unique challenges compared to traditional supports for older adults, when considering the kinds of expertise and systems adjustment required.

There are considerable implications for the patient when mHealth solutions are deployed within the context of health care. Researchers noted end-user concerns about implementing solutions in health care related to the idea of technology replacing, rather than supporting, human contact [73]. Accordingly, developers and health care providers must be sensitive to the needs and preferences of the patient and design solutions [54]. Patients, particularly older adults, have various levels of interest or literacy in technology; consequently, technology support needs to be factored into implementation plans [56]. In other words, mHealth is not a “one size fits all” approach.

Finally, there are policy implications at a population level. Mobile health has the potential to gather large amounts of health data that can be used to better inform interventions and care plans. However, there are many barriers to implementation and sustainability that limit the number of successful, evidence-based mHealth solutions that are implemented beyond the pilot or feasibility stage. For example, the additional costs of privacy / security testing, ongoing technology support/development, and software maintenance are a poor fit with government-supported funding cycles for research and development, where funds are typically delivered for a limited number of months or years [46].

### Implications for Future Research

While this scoping review highlights a number of key design principles and lessons learned for the development and implementation of mHealth solutions, there remain a number of gaps in the literature that should be addressed within future research priorities. First, there is little focus on sustainability of mHealth solutions, and few resources available for researchers to access when navigating the options and planning a sustainability plan. To address this, there may be opportunities for partnerships between industry and research to support the sustainability of an mHealth solution [46]. Second, there are few resources to support evaluating the long-term effect of using mHealth solutions. While the goals and objectives of each solution will vary, researchers would benefit from a theoretical framework to guide the cyclical, iterative process of design, implementation, and evaluation of mHealth technologies as whole entities rather than segmented parts. To this end, longer-term cohort studies and other research designs are needed that can attribute health outcomes to mHealth interventions within complex systems of health care. From a fiscal perspective, studies need to be designed that take into account the cost-effectiveness of new technologies [79].

Researchers also need to consider the unanticipated consequences and risks of mHealth solutions, as well as the potential inequities that may be created given unequal access and use of technologies in society. Potential risks to be considered include the following: (1) the potential for breaches in patient privacy and confidentiality [67,71], (2) the potential for mHealth solutions to replace rather than supplement clinical care [52], and (3) insufficient support or supervision for technologically assisted home-care rehabilitation [81]. It is critical that these risks and concerns, along with those yet unrecognized, are identified in order to manage them appropriately when any new intervention, and particularly technology-based interventions are implemented.

There is limited knowledge about the implementation science related to the adoption and acceptance of new technology in relation to home-based care for older adults. Researchers would benefit from a framework to evaluate the effectiveness of the process of implementing the mHealth solution. For example, where/when/how is support required by patients and their caregivers and how is this best addressed? More focused consideration needs to be given to patient empowerment using mHealth technology for self-management [88,89]. Further, researchers need to anticipate changing needs with different forms of technological experiences within an aging population. Investigators should consider how mHealth solutions targeted for older adults will need to evolve over time as age cohorts and technologies advance.

### Limitations

Various limitations concerning this review need to be considered. First, true to scoping review methods, a quality assessment of selected papers was not used to exclude articles, although all were peer-reviewed. Results from studies using a variety of study designs as well as author opinions were incorporated into the findings of this review. Second, mHealth is a rapidly advancing field. Application of the reported findings may need to be reappraised within the context of a changing landscape of innovation. Third, this scoping review addresses a broad area of content and contexts, that is, different mHealth solutions, goals, and implementation contexts; multiple applications; different users, communities, and countries; and different chronic conditions with rare separation of single conditions from the context of multiple chronic conditions. This may limit transferability of the results to a specific context and present as prime areas for future systematic and realist reviews. Fourth, grey literature is not included; sources for this review were limited to articles published in peer-reviewed journals. Unpublished yet related information on the most current trends in this field may have been missed.

### Conclusions

Developing effective mobile technologies with minimized risk to the quality of health care offered to older adults is a current research priority. Despite the potential benefits that mHealth solutions could offer, there is limited use of these technologies in the home. Interdisciplinary mHealth development teams need to consider specific factors when designing, implementing, and evaluating such technologies that will ultimately fit within the unique context of older adults at home and their care providers. Whether the target of mHealth solutions is the patient, family and/or HCPs, it is imperative to be working *with* these end-users rather than *for* them when designing, implementing, or evaluating mHealth solutions. Optimally, the cyclical and iterative process of mHealth development needs to be viewed as a whole with supportive frameworks to foster this.

The question and selection criteria for this review allowed for a broad range of mHealth technologies to be considered that apply to a variety of chronic conditions associated with aging. This paper presents some commonalities across these different contexts using thematic constructs to inform interconnected processes of design, implementation, and evaluation when developing mHealth solutions best suited to the needs of older adults living at home. At a time of rapid technological innovation, guidelines for research and development in mHealth need to be adaptable to continuous change as new tools become available [90], even as the health care delivery system itself experiences transitions toward community care. With the development of effective and efficient evidence-based technologies, mHealth solutions offer great potential for optimizing the health of an aging population.

## Abbreviations

COPD: chronic obstructive pulmonary disease
CVD: cardiovascular disease
HCP: health care provider
mHealth: mobile health
RCT: randomized controlled trial
SMS: short message service
WHO: World Health Organization
